# Supplementary material for: Novel insights from the Plasmodium falciparum sporozoite-specific proteome by probabilistic integration of 26 studies
Source: PLoS Comput Biol. 2021 Apr 30;17(4):e1008067. doi: 10.1371/journal.pcbi.1008067 (PMC8115857; doi:10.1371/journal.pcbi.1008067)
Supplement: S1 Table — (DOCX) [file pcbi.1008067.s001.docx]

**Table S1**: **Overview of all datasets used for the Bayesian data integration**

Pf = *Plasmodium falciparum*, Pv = *P. vivax*, Pc = *P. cynomolgi*, Py = *P. yoelii*

| **Code** | **Species** | **Type** | **Life stage** | **Reference** |
| --- | --- | --- | --- | --- |
| BA2010 | Pf | Transcriptomics | Asexual | Bartfai *et al.*[1] |
| BU2013 | Pf | Transcriptomics | Asexual | Bunnik *et al.*[2] |
| CA2013 | Pf | Transcriptomics | Asexual, sporozoites | Unpublished, GEO accession GSE52867 |
| EL2016 | Pf | Proteomics | sporozoites | El-Manzalawy *et al.* [3] |
| FL2002 | Pf | Proteomics | Asexual, sporozoites | Florens *et al.*[4] |
| FL2004 | Pf | Proteomics | Asexual | Florens *et al.*[5] |
| LA2008 | Pf | Proteomics | Oocyst, sporozoites | Lasonder *et al.*[6] |
| LA2012 | Pf | Proteomics | Asexual | Lasonder *et al.*[7] |
| LA2015 | Pf | Proteomics | Merozoites | Lasonder *et al.*[8] |
| LA2016 | Pf | Transcriptomics | Gametocytes | Lasonder *et al.*[9] |
| LE2004 | Pf | Transcriptomics & Proteomics | Asexual, gametocyte, sporozoite | Le Roch *et al.*[10, 11] |
| LI2013 | Pf | Proteomics | Salivary gland sporozoite | [12]Lindner et al. |
| LL2006 | Pf | Transcriptomics | Asexual | Llinas *et al.[*13] |
| LO2011 | Pf | Transcriptomics | Asexual | Lopez-Barragan *et al*.[14] |
| MI2017 | Pf | Proteomics | Gametocyte | Miao *et al.*[15] |
| NI2018 | Pf | Proteomics | Asexual | Nilsson *et al.*[16] |
| OE2012 | Pf | Transcriptomics | Asexual | Oehring *et al.*[17] |
| PE2015 | Pf | Transcriptomics | Asexual | Pelle *et al.*[18] |
| RO2017 | Pc | Transcriptomics | Asexual, Liver | Cubi *et al.*[19] |
| SI2008 | Pf | Transcriptomics | Sporozoites | Siau *et al.*[20] |
| SI2010 | Pf | Proteomics | Asexual | Silvestrini *et al.*[21] |
| SW2016 | Pf | Proteomics | Sporozoites | Swearingen *et al*.[22] |
| SW2017 | Pv | Proteomics | Sporozoites | Swearingen *et al*.[23] |
| TA2008 | Py | Proteomics | Liver | Tarun *et al.*[24] |
| WE2010 | Pv | Transcriptomics | Sporozoites | Westenberger *et al*.[25] |
| ZA2018 | Pf | Transcriptomics | Asexual, Sporozoites, Oocyst | Zanghi *et al*.[26] |
| ZH2008 | Pf | Transcriptomics | Asexual, Zygote, Ookinete, Sporozoite | Zhou *et al*.[27] |

1. Bartfai, R., et al., *H2A.Z Demarcates Intergenic Regions of the Plasmodium falciparum Epigenome That Are Dynamically Marked by H3K9ac and H3K4me3.* Plos Pathogens, 2010. **6**(12): p. 14.

2. Bunnik, E.M., et al., *Polysome profiling reveals translational control of gene expression in the human malaria parasite Plasmodium falciparum.* Genome Biology, 2013. **14**(11): p. 18.

3. El-Manzalawy, Y., et al., *PlasmoSEP: Predicting surface-exposed proteins on the malaria parasite using semisupervised self-training and expert-annotated data.* Proteomics, 2016. **16**(23): p. 2967-2976.

4. Florens, L., et al., *A proteomic view of the Plasmodium falciparum life cycle.* Nature, 2002. **419**(6906): p. 520-526.

5. Florens, L., et al., *Proteomics approach reveals novel proteins on the surface of malaria-infected erythrocytes.* Molecular and Biochemical Parasitology, 2004. **135**(1): p. 1-11.

6. Lasonder, E., et al., *Proteomic Profiling of Plasmodium Sporozoite Maturation Identifies New Proteins Essential for Parasite Development and Infectivity.* Plos Pathogens, 2008. **4**(10): p. 18.

7. Lasonder, E., et al., *The Plasmodium falciparum Schizont Phosphoproteome Reveals Extensive Phosphatidylinositol and cAMP-Protein Kinase A Signaling.* Journal of Proteome Research, 2012. **11**(11): p. 5323-5337.

8. Lasonder, E., et al., *Extensive differential protein phosphorylation as intraerythrocytic Plasmodium falciparum schizonts develop into extracellular invasive merozoites.* Proteomics, 2015. **15**(15): p. 2716-2729.

9. Lasonder, E., et al., *Integrated transcriptomic and proteomic analyses of P. falciparum gametocytes: molecular insight into sex-specific processes and translational repression.* Nucleic Acids Research, 2016. **44**(13): p. 6087-6101.

10. Le Roch, K.G., et al., *Discovery of gene function by expression profiling of the malaria parasite life cycle.* Science, 2003. **301**(5639): p. 1503-1508.

11. Le Roch, K.G., et al., *Global analysis of transcript and protein levels across the Plasmodium falciparum life cycle.* Genome Research, 2004. **14**(11): p. 2308-2318.

12. Lindner, S.E., et al., *Total and putative surface proteomics of malaria parasite salivary gland sporozoites.* Mol Cell Proteomics, 2013. **12**(5): p. 1127-43.

13. Llinas, M., et al., *Comparative whole genome transcriptome analysis of three Plasmodium falciparum strains.* Nucleic Acids Research, 2006. **34**(4): p. 1166-1173.

14. Lopez-Barragan, M.J., et al., *Directional gene expression and antisense transcripts in sexual and asexual stages of Plasmodium falciparum.* Bmc Genomics, 2011. **12**: p. 13.

15. Miao, J., et al., *Sex-Specific Biology of the Human Malaria Parasite Revealed from the Proteomes of Mature Male and Female Gametocytes.* Molecular & Cellular Proteomics, 2017. **16**(4): p. 537-551.

16. Nilsson, S.K., et al., *Quantitative Proteomic Profiling Reveals Novel Plasmodium falciparum Surface Antigens and Possible Vaccine Candidates.* Molecular & Cellular Proteomics, 2018. **17**(1): p. 57-74.

17. Oehring, S.C., et al., *Organellar proteomics reveals hundreds of novel nuclear proteins in the malaria parasite Plasmodium falciparum.* Genome Biology, 2012. **13**(11): p. 21.

18. Pelle, K.G., et al., *Transcriptional profiling defines dynamics of parasite tissue sequestration during malaria infection.* Genome Medicine, 2015. **7**: p. 20.

19. Cubi, R., et al., *Laser capture microdissection enables transcriptomic analysis of dividing and quiescent liver stages of Plasmodium relapsing species.* Cellular Microbiology, 2017. **19**(8): p. 9.

20. Siau, A., et al., *Temperature shift and host cell contact up-regulate sporozoite expression of Plasmodium falciparum genes involved in hepatocyte infection.* Plos Pathogens, 2008. **4**(8): p. 13.

21. Silvestrini, F., et al., *Protein Export Marks the Early Phase of Gametocytogenesis of the Human Malaria Parasite Plasmodium falciparum.* Molecular & Cellular Proteomics, 2010. **9**(7): p. 1437-1448.

22. Swearingen, K.E., et al., *Interrogating the Plasmodium Sporozoite Surface: Identification of Surface-Exposed Proteins and Demonstration of Glycosylation on CSP and TRAP by Mass Spectrometry-Based Proteomics.* Plos Pathogens, 2016. **12**(4): p. 32.

23. Swearingen, K.E., et al., *Proteogenomic analysis of the total and surface-exposed proteomes of Plasmodium vivax salivary gland sporozoites.* Plos Neglected Tropical Diseases, 2017. **11**(7): p. 36.

24. Tarun, A.S., et al., *A combined transcriptome and proteome survey of malaria parasite liver stages.* Proceedings of the National Academy of Sciences of the United States of America, 2008. **105**(1): p. 305-310.

25. Westenberger, S.J., et al., *A Systems-Based Analysis of Plasmodium vivax Lifecycle Transcription from Human to Mosquito.* Plos Neglected Tropical Diseases, 2010. **4**(4): p. 17.

26. Zanghi, G., et al., *A Specific PfEMP1 Is Expressed in P. falciparum Sporozoites and Plays a Role in Hepatocyte Infection.* Cell Reports, 2018. **22**(11): p. 2951-2963.

27. Zhou, Y.Y., et al., *Evidence-Based Annotation of the Malaria Parasite's Genome Using Comparative Expression Profiling.* Plos One, 2008. **3**(2): p. 16.
