# Supplementary material for: Novel insights from the Plasmodium falciparum sporozoite-specific proteome by probabilistic integration of 26 studies
Source: PLoS Comput Biol. 2021 Apr 30;17(4):e1008067. doi: 10.1371/journal.pcbi.1008067 (PMC8115857; doi:10.1371/journal.pcbi.1008067)
Supplement: S2 Table — Evidence for expression in other life stages than sporozoites. (DOCX) [file pcbi.1008067.s002.docx]

**Table S2: Negative gold standard members**

Evidence for expression in other life stages

| **Gene ID** | **Name** | **Evidence** |
| --- | --- | --- |
| PF3D7_0102200 | Ring-infected erythrocyte surface antigen | Tiburcia *et al.*[1] |
| PF3D7_0102500 | Erythrocyte antigen-181 | Gilberger *et al.*[2] |
| PF3D7_0109100 | LCCL domain-containing protein | Meerstein-Kessel *et al.*[3] |
| PF3D7_0115100 | Plasmodium exported protein (PHISTa) | Meerstein-Kessel *et al.*[3] |
| PF3D7_0206800 | Merozoite surface protein 2 | Boyle *et al.*[4] |
| PF3D7_0208900 | 6-cysteine protein P230p | Meerstein-Kessel *et al.*[3] |
| PF3D7_0209000 | 6-cysteine protein | Meerstein-Kessel *et al.*[3] |
| PF3D7_0215800 | Origin recognition complex subunit 5 | Gupta *et al.*[5] |
| PF3D7_0219100 | Unknown function protein | Meerstein-Kessel *et al.*[3] |
| PF3D7_0310300 | Phosphoglycerate mutase, putative | Meerstein-Kessel *et al.*[3] |
| PF3D7_0315200 | Circumsporozoite- and TRAP-related protein | Templeton *et al.*[6] |
| PF3D7_0406200 | Sexual stage-specific protein precursor | Meerstein-Kessel *et al.*[3] |
| PF3D7_0415600 | GTP:AMP phosphotransferase | Tewari *et al*.[7] |
| PF3D7_0424200 | Reticulocyte binding protein homologue 4 | Reiling *et al.*[8] |
| PF3D7_0513700 | Secreted ookinete protein, putative | Meerstein-Kessel *et al.*[3] |
| PF3D7_0518700 | mRNA-binding protein PUF1 | Meerstein-Kessel *et al.*[3] |
| PF3D7_0525900 | NIMA related kinase 2 | Meerstein-Kessel *et al.*[3] |
| PF3D7_0605600 | Nucleoside diphosphate kinase, putative | Meerstein-Kessel *et al.*[3] |
| PF3D7_0608600 | Unknown function protein | Meerstein-Kessel *et al.*[3] |
| PF3D7_0705300 | Origin recognition complex subunit 2 | Sharman *et al.*[9] |
| PF3D7_0714100 | MAATS1 domain-containing protein, putative | Meerstein-Kessel *et al.*[3] |
| PF3D7_0719200 | NIMA related kinase 4 | Meerstein-Kessel *et al.*[3] |
| PF3D7_0731500 | Erythrocyte binding antigen-175 | Sim *et al.*[10] |
| PF3D7_0801300 | Von Willebrand factor A domain-related protein | Yuda *et al.*[11] |
| PF3D7_0825900 | Unknown function protein | Meerstein-Kessel *et al.*[3] |
| PF3D7_0930300 | Merozoite surface protein 1 | Hall *et al.*[12] |
| PF3D7_0936600 | Gametocyte exported protein 5 | Tiburcia *et al.*[1] |
| PF3D7_1028700 | Merozoite TRAP-like protein | Baum *et al.*[13] |
| PF3D7_1031000 | Ookinete surface protein P25 | Ngwa *et al.*[14] |
| PF3D7_1035700 | Duffy binding-like merozoite surface protein | Wickramarachchi *et al.*[15] |
| PF3D7_1036300 | Duffy binding-like merozoite surface protein 2 | Hodder *et al.*[16] |
| PF3D7_1038400 | Gametocyte-specific protein | Meerstein-Kessel *et al.*[3] |
| PF3D7_1104600 | Radial spoke head protein, putative | Meerstein-Kessel *et al.*[3] |
| PF3D7_1121800 | Peptidase M16, putative | Meerstein-Kessel *et al.*[3] |
| PF3D7_1128300 | ATP-dependent 6-phosphofructokinase | Meerstein-Kessel *et al.*[3] |
| PF3D7_1203000 | Origin recognition complex subunit 1 | Deshmukh *et al.*[17] |
| PF3D7_1311800 | M1-family alanyl aminopeptidase | Azimzadeh *et al.*[18] |
| PF3D7_1361900 | Proliferating cell nuclear antigen 1 | Mitra *et al.*[19] |
| PF3D7_1457000 | Signal peptide peptidase | Maraoana *et al.*[20] |

1. Tiburcio, M., et al., *Specific expression and export of the Plasmodium falciparum Gametocyte EXported Protein-5 marks the gametocyte ring stage.* Malaria Journal, 2015. **14**: p. 12.

2. Gilberger, T.W., et al., *A novel erythrocyte binding antigen-175 paralogue from Plasmodium falciparum defines a new trypsin-resistant receptor on human erythrocytes.* Journal of Biological Chemistry, 2003. **278**(16): p. 14480-14486.

3. Meerstein-Kessel, L., et al., *Probabilistic data integration identifies reliable gametocyte-specific proteins and transcripts in malaria parasites.* Scientific Reports, 2018. **8**: p. 13.

4. Boyle, M.J., et al., *Sequential Processing of Merozoite Surface Proteins during and after Erythrocyte Invasion by Plasmodium falciparum.* Infection and Immunity, 2014. **82**(3): p. 924-936.

5. Gupta, A., P. Mehra, and S.K. Dhar, *Plasmodium falciparum origin recognition complex subunit 5: functional characterization and role in DNA replication foci formation.* Molecular Microbiology, 2008. **69**(3): p. 646-665.

6. Templeton, T.J., D.C. Kaslow, and D.A. Fidock, *Developmental arrest of the human malaria parasite Plasmodium falciparum within the mosquito midgut via CTRP gene disruption.* Molecular Microbiology, 2000. **36**(1): p. 1-9.

7. Tewari, R., et al., *The systematic functional analysis of Plasmodium protein kinases identifies essential regulators of mosquito transmission.* Cell Host Microbe, 2010. **8**(4): p. 377-87.

8. Reiling, L., et al., *The Plasmodium falciparum erythrocyte invasion ligand Pfrh4 as a target of functional and protective human antibodies against malaria.* PLoS One, 2012. **7**(9): p. e45253.

9. Sharma, R., et al., *Identification of a novel trafficking pathway exporting a replication protein, Orc2 to nucleus via classical secretory pathway in &ITPlasmodium falciparum&IT.* Biochimica Et Biophysica Acta-Molecular Cell Research, 2018. **1865**(5): p. 817-829.

10. Sim, B.K.L., et al., *Delineation of Stage Specific Expression of Plasmodium falciparum EBA-175 by Biologically Functional Region II Monoclonal Antibodies.* Plos One, 2011. **6**(4): p. 10.

11. Yuda, M., et al., *von Willebrand Factor A Domain-related Protein, a novel microneme protein of the malaria ookinete highly conserved throughout Plasmodium parasites.* Molecular and Biochemical Parasitology, 2001. **116**(1): p. 65-72.

12. Hall, R., et al., *PROCESSING, POLYMORPHISM, AND BIOLOGICAL SIGNIFICANCE OF P190, A MAJOR SURFACE-ANTIGEN OF THE ERYTHROCYTIC FORMS OF PLASMODIUM-FALCIPARUM.* Molecular and Biochemical Parasitology, 1984. **11**(APR): p. 61-80.

13. Baum, J., et al., *A conserved molecular motor drives cell invasion and gliding motility across malaria life cycle stages and other apicomplexan parasites.* Journal of Biological Chemistry, 2006. **281**(8): p. 5197-5208.

14. Ngwa, C.J., et al., *Changes in the transcriptome of the malaria parasite Plasmodium falciparum during the initial phase of transmission from the human to the mosquito.* Bmc Genomics, 2013. **14**: p. 21.

15. Wickramarachchi, T., et al., *A novel Plasmodium falciparum erythrocyte binding protein associated with the merozoite surface, PfDBLMSP.* International Journal for Parasitology, 2009. **39**(7): p. 763-773.

16. Hodder, A.N., et al., *Insights into Duffy Binding-like Domains through the Crystal Structure and Function of the Merozoite Surface Protein MSPDBL2 from Plasmodium falciparum.* Journal of Biological Chemistry, 2012. **287**(39): p. 32922-32939.

17. Deshmukh, A.S., et al., *The role of N-terminus of Plasmodium falciparum ORC1 in telomeric localization and var gene silencing.* Nucleic Acids Research, 2012. **40**(12): p. 5313-5331.

18. Azimzadeh, O., et al., *Plasmodium falciparum PfA-M1 aminopeptidase is trafficked via the parasitophorous vacuole and marginally delivered to the food vacuole.* Malaria Journal, 2010. **9**: p. 16.

19. Mitra, P., et al., *Functional dissection of proliferating-cell nuclear antigens (1 and 2) in human malarial parasite Plasmodium falciparum: possible involvement in DNA replication and DNA damage response.* Biochemical Journal, 2015. **470**: p. 115-129.

20. Marapana, D.S., et al., *Malaria Parasite Signal Peptide Peptidase is an ER-Resident Protease Required for Growth but not for Invasion.* Traffic, 2012. **13**(11): p. 1457-1465.
