# Supplementary material for: Novel insights from the Plasmodium falciparum sporozoite-specific proteome by probabilistic integration of 26 studies
Source: PLoS Comput Biol. 2021 Apr 30;17(4):e1008067. doi: 10.1371/journal.pcbi.1008067 (PMC8115857; doi:10.1371/journal.pcbi.1008067)
Supplement: S4 Table — (DOCX) [file pcbi.1008067.s004.docx]

**Table S4:** **Sporozoite enriched proteins annotated as “unknown function” in PlasmoDB for which orthology with human proteins could be detected using best bidirectional hits at the level of sequence profiles**

|  | Human Gene symbol | E value |
| --- | --- | --- |
| PF3D7_1320300 | SMPD2 | 2.1E-28 |
| PF3D7_1304600 | SETD3 | 3.9E-27 |
| PF3D7_1112800 | KIAA0895 | 7.4E-69 |
| PF3D7_1359700 | SACS | 7.9E-35 |
| PF3D7_1329200 | C22ORF23 | 0.0000025 |
| PF3D7_1432600 | XPC | 7.9E-94 |
| PF3D7_0726600 | FAM13A | 9.6E-43 |
| PF3D7_1351300 | STIMATE | 1.7E-60 |
| PF3D7_1142200 | IST1 | 2.2E-49 |
| PF3D7_0301900 | APTX | 4.9E-20 |
| PF3D7_1357600 | MRPL35 | 6.5E-22 |
| PF3D7_1313600 | CLUH | 3.7E-49 |
| PF3D7_0503900 | PHYH | 7E-27 |
| PF3D7_1309900 | SDHAF2 | 3.6E-24 |
| PF3D7_1367400 | NOP9 | 5E-67 |
| PF3D7_1238200 | TWF2 | 8.6E-52 |
| PF3D7_0209400 | ARMT1 | 1E-110 |
| PF3D7_1313500 | PKD2L2 | 7.7E-09 |
| PF3D7_1145800 | POLR3C | 2.5E-48 |
| PF3D7_0911400 | NAA35 | 5.7E-34 |
| PF3D7_1322600 | PLCXD2 | 2.4E-34 |
| PF3D7_0531400 | TFIP11 | 3.5E-53 |
| PF3D7_1246000 | IFI30 | 1.1E-22 |
| PF3D7_1473900 | NUFI1P | 3.9E-35 |
| PF3D7_1303300 | ABITRAM | 6.5E-37 |
| PF3D7_1439200 | DNAAF4 | 1.4E-47 |
| PF3D7_0718800 | RBPP9 | 2E-16 |
