# Supplementary material for: Novel insights from the Plasmodium falciparum sporozoite-specific proteome by probabilistic integration of 26 studies
Source: PLoS Comput Biol. 2021 Apr 30;17(4):e1008067. doi: 10.1371/journal.pcbi.1008067 (PMC8115857; doi:10.1371/journal.pcbi.1008067)
Supplement: S5 Table — Enrichment based on the relative absence of the proteins involved at an FDR < = 0.01 and an enrichment score > 0.20. The type II Fatty Acid Synthesis are the genes from Fig 3, of which the gold standard genes were left out in the GSEA analysis. The “translocation of peptides or proteins into hosts” GO category did not have any proteins among the sporozoite enriched proteins, and was left out of the description of the results. (N)ES: (normalized) enrichment score, FDR: false discovery rate. (DOCX) [file pcbi.1008067.s005.docx]

**Table S5**: **Biological processes enriched (top list) or depleted (bottom list) in the sporozoite**

Enrichment based on the relative absence of the proteins involved at an FDR <= 0.01 and an enrichment score > 0.20. The type II Fatty Acid Synthesis are the genes from Figure 3, of which the gold standard genes were left out in the GSEA analysis. The “translocation of peptides or proteins into hosts” GO category did not have any proteins among the sporozoite enriched proteins, and was left out of the description of the results. (N)ES: (normalized) enrichment score, FDR: false discovery rate

**Enriched**

| **GS details** | **ES** | **NES** | **NOM p-val** | **FDR q-val** |
| --- | --- | --- | --- | --- |
| Type II Fatty Acid synthesis[72] | 0.53 | 2.39 | 0.000 | 0.003 |
| [GO:0042000](http://amigo.geneontology.org/amigo/term/GO:0042000)  translocation of peptides or proteins into host | 0.62 | 2.36 | 0.000 | 0.003 |
| [GO:0006633](http://amigo.geneontology.org/amigo/term/GO:0006633)  Fatty acid biosynthesis | 0.47 | 2.31 | 0.000 | 0.004 |
| [GO:0006506](http://amigo.geneontology.org/amigo/term/GO:0006506) GPI anchor biosynthesis | 0.52 | 2.12 | 0.002 | 0.010 |

**Depleted**

| **GS DETAILS** | **ES** | **NES** | **NOM p-val** | **FDR q-val** |
| --- | --- | --- | --- | --- |
| [GO:0006511](http://amigo.geneontology.org/amigo/term/GO:0006511)ubiquitin-dependent protein catabolic process | -0.53 | -4.62 | 0.000 | 0.000 |
| [GO:0006412](http://amigo.geneontology.org/amigo/term/GO:0006412) translation | -0.29 | -4.21 | 0.000 | 0.000 |
| [GO:0042493](http://amigo.geneontology.org/amigo/term/GO:0042493) response to drug | -0.30 | -3.37 | 0.000 | 0.000 |
| [GO:0051603](http://amigo.geneontology.org/amigo/term/GO:0051603) proteolysis involved in cellular protein catabolic process | -0.77 | -3.71 | 0.000 | 0.000 |
| [GO:0006457](http://amigo.geneontology.org/amigo/term/GO:0006457) protein folding | -0.38 | -3.58 | 0.000 | 0.000 |
| [GO:0006888](http://amigo.geneontology.org/amigo/term/GO:0006888) endoplasmic reticulum to Golgi vesicle-mediated transport | -0.55 | -2.89 | 0.000 | 0.000 |
| [GO:0006096](http://amigo.geneontology.org/amigo/term/GO:0006096) glycolytic process | -0.62 | -3.02 | 0.000 | 0.000 |
| [GO:0055114](http://amigo.geneontology.org/amigo/term/GO:0055114) oxidation-reduction process | -0.26 | -2.50 | 0.000 | 0.001 |
| [GO:0009408](http://amigo.geneontology.org/amigo/term/GO:0009408) response to heat | -0.50 | -2.66 | 0.000 | 0.000 |
| [GO:0000398](http://amigo.geneontology.org/amigo/term/GO:0000398) mRNA splicing, via spliceosome | -0.32 | -2.67 | 0.000 | 0.002 |
| GO:0060627 regulation of vesicle-mediated transport | -0.63 | -2.40 | 0.000 | 0.001 |
| [GO:0006886](http://amigo.geneontology.org/amigo/term/GO:0006886) intracellular protein transport | -0.27 | -2.50 | 0.000 | 0.001 |
| [GO:0008380](http://amigo.geneontology.org/amigo/term/GO:0008380) RNA splicing | -0.47 | -2.45 | 0.000 | 0.000 |
| [GO:0006413](http://amigo.geneontology.org/amigo/term/GO:0006413) translation initiation | -0.37 | -2.40 | 0.000 | 0.002 |
| [GO:0006099](http://amigo.geneontology.org/amigo/term/GO:0006099) tricarboxylic acid cycle | -0.45 | -2.35 | 0.000 | 0.003 |
| [GO:0006260](http://amigo.geneontology.org/amigo/term/GO:0006099) DNA replication | -0.45 | -2.35 | 0.000 | 0.001 |
| GO:0006986 response to unfolded protein | -0.54 | -2.32 | 0.000 | 0.002 |
| GO:0015986 ATP synthesis coupled proton transport | -0.47 | -2.13 | 0.000 | 0.006 |
