# Supplementary material for: Novel insights from the Plasmodium falciparum sporozoite-specific proteome by probabilistic integration of 26 studies
Source: PLoS Comput Biol. 2021 Apr 30;17(4):e1008067. doi: 10.1371/journal.pcbi.1008067 (PMC8115857; doi:10.1371/journal.pcbi.1008067)
Supplement: S7 Table — Information for all annotated Plasmodium falciparum proteins and the proteins considered immunogenic and sequencing depth. (DOCX) [file pcbi.1008067.s007.docx]

**S8 Table: Levels of polymorphism in NF54, NF135 and NF166 relative of the reference strain 3D7**. Information for all annotated Plasmodium falciparum proteins and the proteins considered immunogenic and sequencing depth.

| **In all proteins** | **NF54** | **NF135** | **NF166** |
| --- | --- | --- | --- |
| **Non-synonymous SNPs** | 27 | 8,046 | 7,062 |
| **Indels** | 9 | 2,638 | 2,620 |
| **All polymorphisms** | 43 | 13,431 | 12,365 |
| **In antigenic proteins** |  | | |
| **Non-synonymous SNPs** | 2 | 1,235 | 2,177 |
| **Indels** | 3 | 742 | 774 |
| **All polymorphisms** | 8 | 2,376 | 3,586 |
| **# of mutated proteins** | 7 | 373 | 378 |
| **Average (median) coverage** |  | | |
| **Coding** | 28.7 (25) | 35.7 (32) | 44.4 (39) |
| **Non-coding** | 16.2 (12) | 20.9 (16) | 23.1 (18) |
