# Supplementary figures and images for: Novel insights from the Plasmodium falciparum sporozoite-specific proteome by probabilistic integration of 26 studies
Source: PLoS Comput Biol. 2021 Apr 30;17(4):e1008067. doi: 10.1371/journal.pcbi.1008067 (PMC8115857; doi:10.1371/journal.pcbi.1008067)

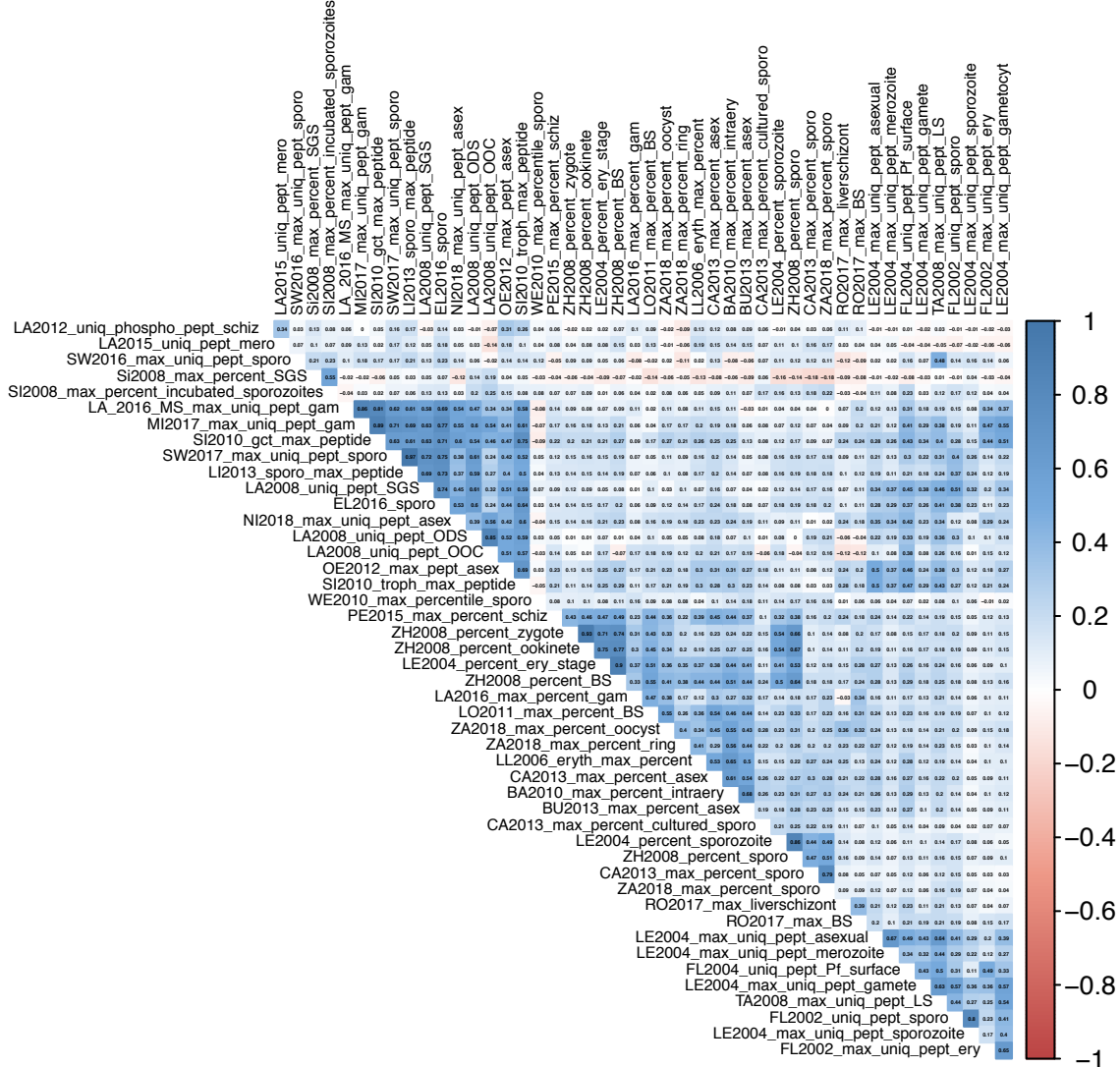

Supplement: S1 Fig — Study code (two letters and year) as in S1 Table. Samples are coded as sporozoite (sporo), salivary gland sporozoite (SGS), oocyst derived sporozoite (ODS) or oocyst (OOC), liver stage (LS), blood stage (BS, ery, asexual, merozoite, schiz, ring) or other stages (gametocyte/gam, zygote, ookinete). Transcriptomics studies are given a percentile (percent) for each gene they detected and proteomics studies a score for each unique peptide (uniq_pept) per gene. (PDF) [file pcbi.1008067.s008.pdf]

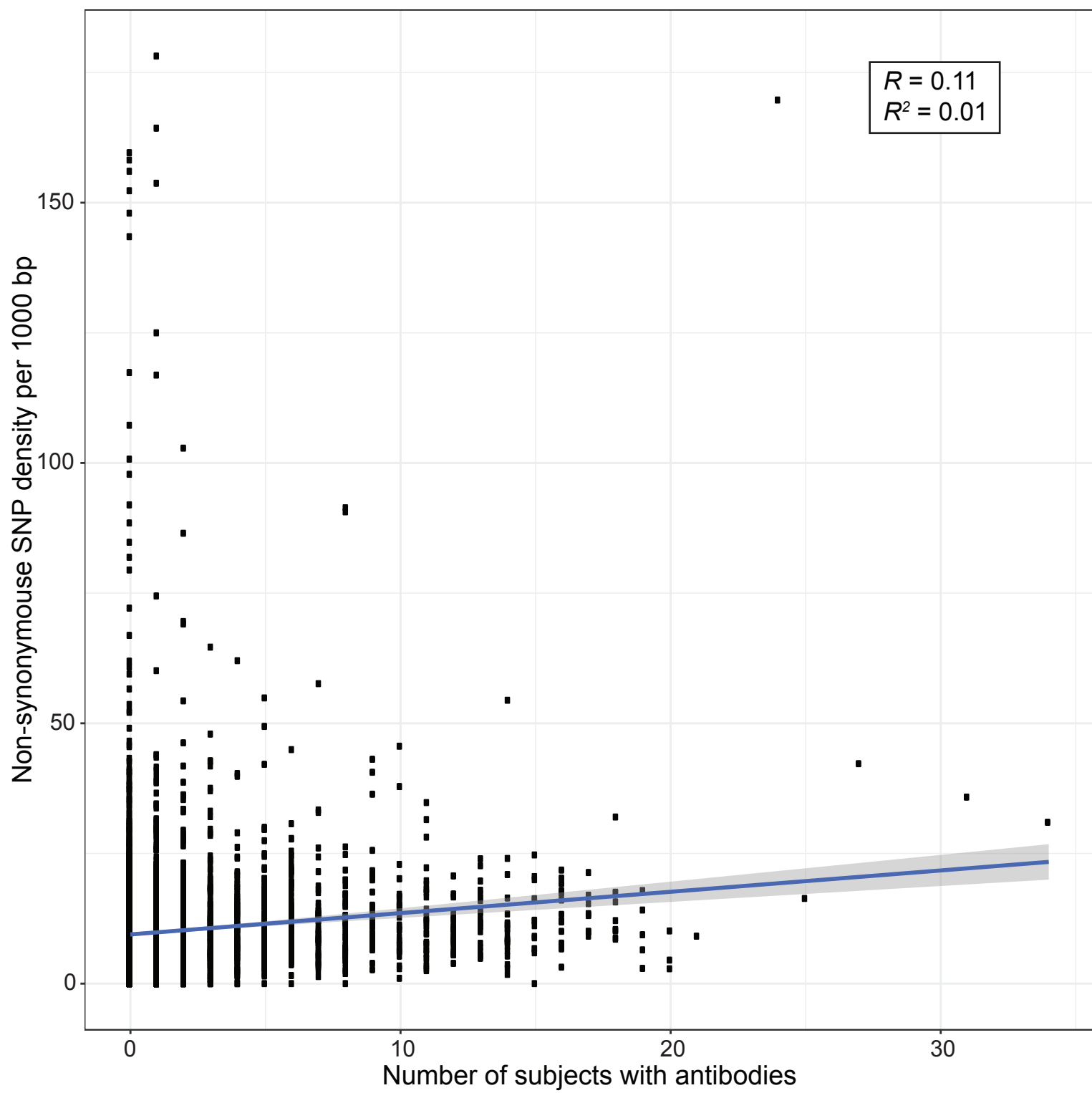

Supplement: S3 Fig — Antibody prevalence shows moderate correlation with the number of Non-synonymous SNPs per kb coding region of the respective gene (PlasmoDB). (PDF) [file pcbi.1008067.s010.pdf]

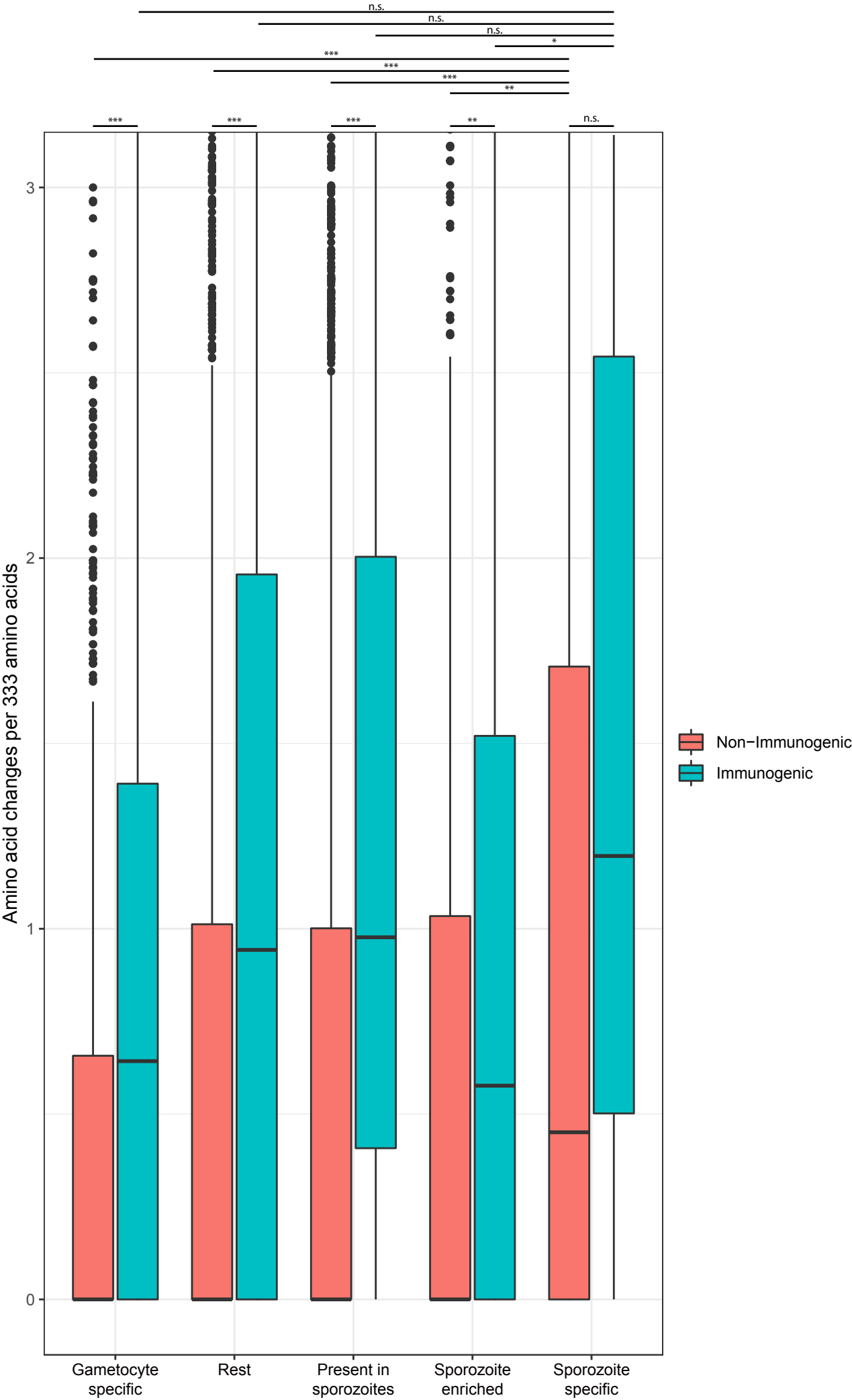

Supplement: S4 Fig — Sporozoite proteins, selected at various levels of stringency, gametocyte proteins, and the remaining proteins. Sporozoite enriched or sporozoite specific proteins show relatively high levels of polymorphisms, while gametocyte proteins are clearly depleted of polymorphisms. Furthermore, antigenic proteins of either stage are enriched in polymorphisms relative to non-antigenic proteins. (PDF) [file pcbi.1008067.s011.pdf]

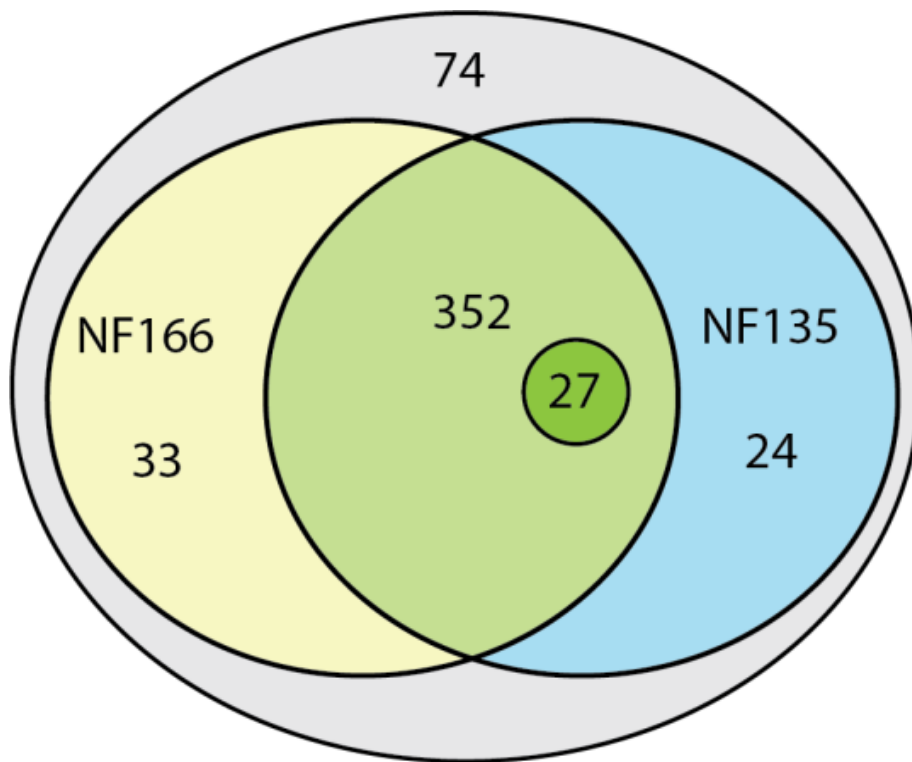

Immunogenic proteins, n=483

Supplement: S5 Fig — SNPs in NF135 (blue) and NF166 (yellow) compared to the reference in NF54/3D7. The grey shaded area contains proteins without any SNPs, they are hence identical to the 3D7 reference and NF54 strain in both NF135 and NF166. The overlap (light green) shows proteins that have SNPs in both NF135 and NF166, and 27 proteins (dark green) have the exact same SNPs in both strains. (PDF) [file pcbi.1008067.s012.pdf]
