# Supplementary material for: Novel insights from the Plasmodium falciparum sporozoite-specific proteome by probabilistic integration of 26 studies
Source: PLoS Comput Biol. 2021 Apr 30;17(4):e1008067. doi: 10.1371/journal.pcbi.1008067 (PMC8115857; doi:10.1371/journal.pcbi.1008067)
Supplement: S2 Fig — The in PF3D7_0104100 predicted transmembrane regions are underlined, the conserved cysteines are boxed. Do note that the fourth transmembrane region is relatively long. Predictions with other tools than TMHMM [95] like Phobius [96] indicate a shorter TM region, putting the cysteine that is located in that TM region in the extracellular space. The Toxoplasma protein was included because its sequence profile has significant sequence similarity against both the human protein profile (E = 2e-20) and the Plasmodium protein profile (E = 3.4e-44), while the similarity between the human and the Plasmodium protein is less significant (E = 0.0001). (PDF) [file pcbi.1008067.s009.pdf]

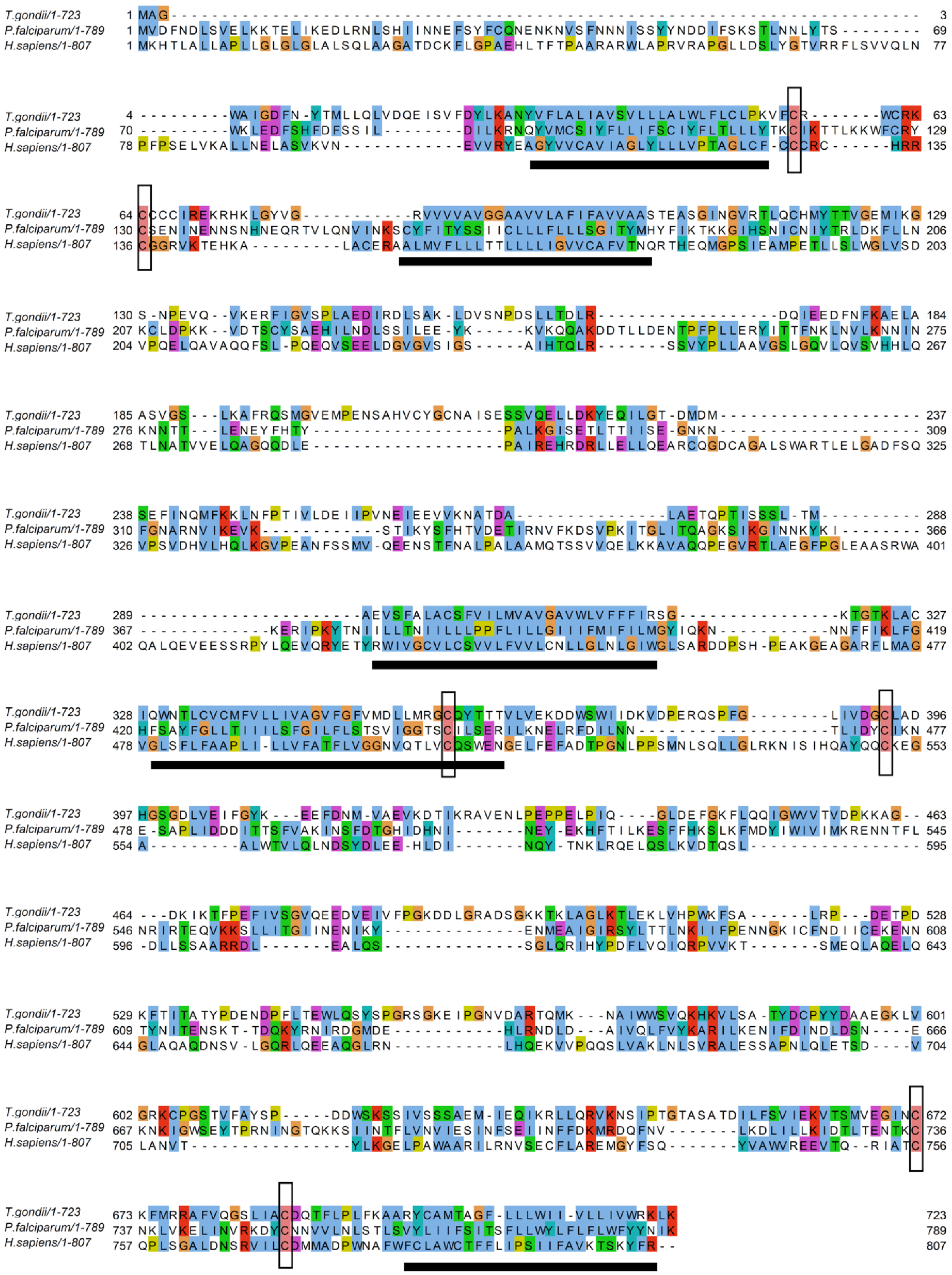

T.gondii/1-723 1 MAG-- 3
P.falciparum/1-789 1 MVDFNDLSVELKKTELIKEDLRNLSHIINNEFSYFCQNENKNVSFNNNISSYYNDDIFSKSTLNNLYTS-------- 69
H.sapiens/1-807 1 MKHTLALLAPLLGLGLGLALSQLAAGATDCKFLGPAEHLTFTPAARARWLAPRVRAPGLLDSLYGTVRRFLSVVQLN 77
T.gondii/1-723 4 ---------WAIGDFN-YTMLLQLVDQEISVFDYLKANYVFLALIAVSVLLLALWLFLCLPKVFCR-------WCRK 63
P.falciparum/1-789 70 ---------WKLEDFSHFDFSSIL--------DILKRNQYVMCSIYFLLIFSCIYFLTLLLYTKCIKTTLKKWFCRY 129
H.sapiens/1-807 78 PFPSELVKALLNELASVKVN------------EVVRYEAGYVVCAVIAGLYLLLVPTAGLCF-CCCRC------HRR 135
T.gondii/1-723 64 CCCCIREKRHKLGYVG-----------RVVVVAVGGAAVVLAFIFAVVAASTEASGINGVRTLQCHMYTTVGEMIKG 129
P.falciparum/1-789 130 CSENINENNSNHNEQRTVLQNVINKSCYFITYSSIICLLLFLLLSGITYMHYFIKTKKGIHSNICNIYTRLDKFLLN 206
H.sapiens/1-807 136 CGGRVKTEHKA---------LACERAALMVFLLLTTLLLLIGVVCAFVTNQRTHEQMGPSIEAMPETLLSLWGLVSD 203
T.gondii/1-723 130 S-NPEVQ--VKERFIGVSPLAEDIRDLSAK-LDVSNPDSLLTDLR------------------DQIEEDFNFKAELA 184
P.falciparum/1-789 207 KCLDPKK--VDTSCYSAEHILNDLSSILEE-YK-----KVKQQAKDDTLLDENTPFPLLERYITTFNKLNVLKNNIN 275
H.sapiens/1-807 204 VPQELQAVAQQFSL-PQEQVSEELDGVGVSIGS-----AIHTQLR-------SSVYPLLAAVGSLGQVLQVSVHHLQ 267
T.gondii/1-723 185 ASVGS---LKAFRQSMGVEMPENSAHVCYGCNAISESSVQELLDKYEQILGT-DMDM-------------------- 237
P.falciparum/1-789 276 KNNTT---LENEYFHTY-------------------PALKGISETLTTIISE-GNKN-------------------- 309
H.sapiens/1-807 268 TLNATVVELQAGQQDLE-------------------PAIREHRDRLLELLQEARCQGDCAGALSWARTLELGADFSQ 325
T.gondii/1-723 238 SEFINQMFKKLNFPTIVLDEIIPVNEIEEVVKNATDA---------------LAETQPTISSSL-TM---------- 288
P.falciparum/1-789 310 FGNARNVIKEVK------------STIKYSFHTVDETIRNVFKDSVPKITGLITQAGKSIKGINNKYKI-------- 366
H.sapiens/1-807 326 VPSVDHVLHQLKGVPEANFSSMV-QEENSTFNALPALAAMQTSSVVQELKKAVAQQPEGVRTLAEGFPGLEAASRWA 401
T.gondii/1-723 289 -----------------------AEVSFALACSFVILMVAVGAVWLVFFFIRSG---------------KTGTKLAC 327
P.falciparum/1-789 367 -------------KERIPKYTNIILLTNIILLLPPFLILLGIIIFMIFILMGYIQKN-----------NNFFIKLFG 419
H.sapiens/1-807 402 QALQEVEESSRPYLQEVQRYETYRWIVGCVLCSVVLFVVLCNLLGLNLGIWGLSARDDPSH-PEAKGEAGARFLMAG 477
T.gondii/1-723 328 IQWNTLCVCMFVLLIVAGVFGFVMDLLMRGCQYTTTVLVEKDDWSWIIDKVDPERQSPFG--------LIVDGCLAD 396
P.falciparum/1-789 420 HFSAYFGLLTIIILSFGILFLSTSVIGGTSCILSERILKNELRFDILNN-------------------TLIDYCIKN 477
H.sapiens/1-807 478 VGLSFLFAAPLI-LLVFATFLVGGNVQTLVCQSWENGELFEFADTPGNLPPSMNLSQLLGLRKNISIHQAYQQCKEG 553
T.gondii/1-723 397 HGSGDLVEIFGYK---EEFDNM-VAEVKDTIKRAVENLPEPPELPIQ----GLDEFGKFLQQIGWVVTVDPKKAG-- 463
P.falciparum/1-789 478 E-SAPLIDDDITTSFVAKINSFDTGHIDHNI-------NEY-EKHFTILKESFFHKSLKFMDYIWIVIMKRENNTFL 545
H.sapiens/1-807 554 A---------ALWTVLQLNDSYDLEE-HLDI-------NQY-TNKLRQELQSLKVDTQSL----------------- 595
T.gondii/1-723 464 ---DKIKTFPEFIVSGVQEEDVEIVFPGKDDLGRADSGKKTKLAGLKTLEKLVHPWKFSA------LRP---DETPD 528
P.falciparum/1-789 546 NRIRTEQVKKSLLITGIINENIKY--------------ENMEAIGIRSYLTTLNKIIFPENNGKICFNDIICEKENN 608
H.sapiens/1-807 596 -DLLSSAARRDL-------EALQS--------------SGLQRIHYPDFLVQIQRPVVKT-------SMEQLAQELQ 643
T.gondii/1-723 529 KFTITATYPDENDPFLTEWLQSYSPGRSGKEIPGNVDARTQMK---NAIWWSVQKHKVLSA-TYDCPYYDAAEGKLV 601
P.falciparum/1-789 609 TYNITENSKT-TDQKYRNIRDGMDE-----------HLRNDLD---AIVQLFVYKARILKENIFDINDLDSN----E 666
H.sapiens/1-807 644 GLAQAQDNSV-LGQRLQEEAQGLRN-----------LHQEKVVPQQSLVAKLNLSVRALESSAPNLQLETSD----V 704
T.gondii/1-723 602 GRKCPGSTVFAYSP-----DDWSKSSIVSSSAEM-IEQIKRLLQRVKNSIPTGTASATDILFSVIEKVTSMVEGINC 672
P.falciparum/1-789 667 KNKIGWSEYTPRNINGTQKKSIINTFLVNVIESINFSEIINFFDKMRDQFNV-------LKDLILLKIDTLTENTKC 736
H.sapiens/1-807 705 LANVT----------------YLKGELPAWAARILRNVSECFLAREMGYFSQ-------YVAWVREEVTQ--RIATC 756
T.gondii/1-723 673 KFMRRAFVQGSLIACDQTFLPLFKAARYCAMTAGF-LLLWII-VLLIVWRKLK 723
P.falciparum/1-789 737 NKLVKELINVRKDYCNNVVLNLSTLSVYLIIFSITSFLLWYLFLFLWFYYNIK 789
H.sapiens/1-807 757 QPLSGALDNSRVILCDMMADPWNAFWFCLAWCTFFLIPSIIFAVKTSKYFR-- 807
